# Physiological Adaptation to Plant-Based Diets: A Homeostatic Framework for Sustained Dietary Change

**DOI:** 10.3390/biology15171520

**Published:** 2026-09-03

**Authors:** Miguel López-Moreno, Matthew Nagra, Mariana Del Carmen Fernández-Fígares Jiménez, Gian Pierre Gomez-Herrera, Hana Kahleova

**Affiliations:** 1 Diet, Planetary Health and Performance, Faculty of Health Sciences, Universidad Francisco de Vitoria, 28223 Madrid, Spain; 2 Institute of Health and Sport Sciences, Faculty of Health Sciences, Universidad Francisco de Vitoria, 28223 Madrid, Spain; 3 Department of Family Practice, University of British Columbia, Vancouver, BC V6T 1Z3, Canada; matthew.nagra@ubc.ca; 4 School of Medicine, University of Granada, 18071 Granada, Spain; marianafigares@correo.ugr.es; 5 Instituto de Investigación Nutricional (IIN), Lima 15000, Peru; gianpierre.gh@gmail.com; 6 Departamento de Nutrición y Dietética, Hospital Regional Hermilio Valdizan Medrano, MINSA, Huánuco 10000, Peru; 7 Physicians Committee for Responsible Medicine, Washington, DC 20016, USA; 8 Diabetes Center, Institute for Clinical and Experimental Medicine, 140 21 Prague, Czech Republic

**Keywords:** plant-based diet, vegan diet, physiological adaptation, iron homeostasis, creatine, protein metabolism, gut microbiota, nutrient bioavailability

## Abstract

Plant-based dietary patterns are typically discussed in terms of nutrients of concern—iron, creatine, indispensable amino acids, and rapidly fermented carbohydrates—based on the assumption that reduced dietary exposure necessarily translates into reduced physiological availability. This review examines physiological adaptation as an alternative framework, drawing on human intervention and observational evidence across five biological systems: iron homeostasis, endogenous biosynthesis of creatine and carnosine, protein metabolism, fermentation of dietary fiber by the gut microbiota, and the microbial biotransformation of plant polyphenols. Sustained changes in dietary exposure are accompanied by coordinated homeostatic responses, including increased intestinal iron absorption, upregulated endogenous synthesis, improved nitrogen economy, functional remodeling of the gut microbiota, and enhanced polyphenol biotransformation, which contribute to maintaining physiological function. However, adaptive capacity is not unlimited: pregnancy, chronic inflammation, malabsorptive disease, and severe or prolonged dietary insufficiency can exceed adaptive capacity and increase the physiological relevance of dietary intake, planning, or supplementation. Recognizing physiological adaptation as a distinct, measurable phenomenon reframes plant-based diets less as inherently deficient patterns requiring correction and more as a physiological model for understanding how the human body responds to sustained dietary change, with implications for interpreting acute versus long-term evidence and for nutritional counseling.

## Simple Summary

Plant-based diets are often discussed in terms of nutrients that may be less available when foods of animal origin are reduced or excluded. However, the human body is not a passive recipient of dietary nutrients: it can adjust to sustained changes in food intake through natural processes that help maintain internal balance. This review examines how these adaptations may occur in people following plant-based diets, focusing on iron, compounds such as creatine and carnosine, protein metabolism, dietary fiber, and polyphenols. The available evidence suggests that long-term dietary changes can trigger increased iron absorption, a greater production of some compounds by the body, more efficient use and recycling of amino acids, changes in the gut bacteria that improve tolerance to fiber, and greater transformation of plant compounds into metabolites that can be used by the body. However, these adaptations have limits. Higher nutritional requirements, chronic inflammation, digestive disorders, severe dietary insufficiency, and other health conditions may reduce the ability of the body to compensate. Understanding these adaptations can improve how plant-based diets are evaluated, help distinguish short-term responses from long-term effects, and support more individualized dietary advice.

## 1. Introduction

The relationship between dietary intake and physiological function is commonly interpreted through an implicit linear paradigm in nutritional science, whereby changes in nutrient intake are expected to produce proportional changes in biological function. This assumption underlies nutrient reference values, dietary recommendations, nutritional epidemiology, and the interpretation of many randomized clinical trials [1,2]. Within this framework, reducing the intake of a nutrient or food component is generally expected to decrease its biological availability, and consequently, impair the physiological processes that depend upon it. Although this approach has been fundamental for understanding nutrient deficiencies and establishing dietary recommendations, it does not fully capture the dynamic physiological responses that occur following sustained changes in dietary exposure.

Physiological responses to dietary change are regulated by homeostatic mechanisms that maintain internal stability despite fluctuations in nutrient availability [3,4]. These mechanisms involve coordinated endocrine, metabolic, intestinal and cellular responses that modify the relationship between dietary exposure and physiological function. Well-established examples include the regulation of glucose, calcium, and iron homeostasis, in which physiological outcomes depend not only on nutrient intake, but also on endogenous mechanisms controlling nutrient absorption, metabolism, storage, and tissue distribution [5,6,7,8,9]. Consequently, dietary exposure alone does not necessarily predict physiological function [10].

Plant-based dietary patterns provide a particularly informative model for studying these adaptive responses because they simultaneously modify exposure to multiple nutrients and bioactive food components. Compared with omnivorous diets, they are characterized by lower exposure to heme iron, creatine, and in some contexts, highly digestible protein, together with higher intakes of dietary fiber, fermentable carbohydrates, and phytochemicals [11,12,13]. Consequently, much of the literature has focused on identifying nutrients of concern, evaluating dietary adequacy, and proposing strategies such as dietary planning, food fortification, or supplementation to ensure nutritional sufficiency [14,15]. Accordingly, discussions surrounding plant-based diets have traditionally emphasized nutritional adequacy and nutrients of concern rather than the physiological adaptations accompanying long-term dietary adherence. Viewing these dietary patterns through a physiological lens may therefore provide broader insights into how humans adapt to sustained alterations in dietary exposure while maintaining nutritional and metabolic homeostasis.

Rather than considering plant-based diets solely in terms of nutrient adequacy, this review examines them as a model of human physiological adaptation, discussing how coordinated homeostatic responses contribute to maintaining nutritional and metabolic function during long-term dietary adherence.

Relevant literature was identified through targeted searches of PubMed, Scopus, and Web of Science, complemented by examination of the reference lists of key publications. The initial search was conducted on 17 June 2026 using combinations of the following keywords: ‘plant-based diet’, ‘vegan diet’, ‘vegetarian diet*’, ‘physiological adaptation’, ‘metabolic adaptation’, ‘homeostasis’, ‘protein metabolism’, ‘iron metabolism’, ‘gut microbiota’, ‘polyphenol*’, ‘creatine’, and ‘carnosine’. The searches focused primarily on human studies examining physiological responses, metabolic adaptation, and long-term outcomes associated with plant-based dietary patterns. Additional studies were considered when they provided relevant mechanistic or physiological evidence to support the proposed framework.

## 2. Physiological Principles of Dietary Adaptation

**Figure 1:**
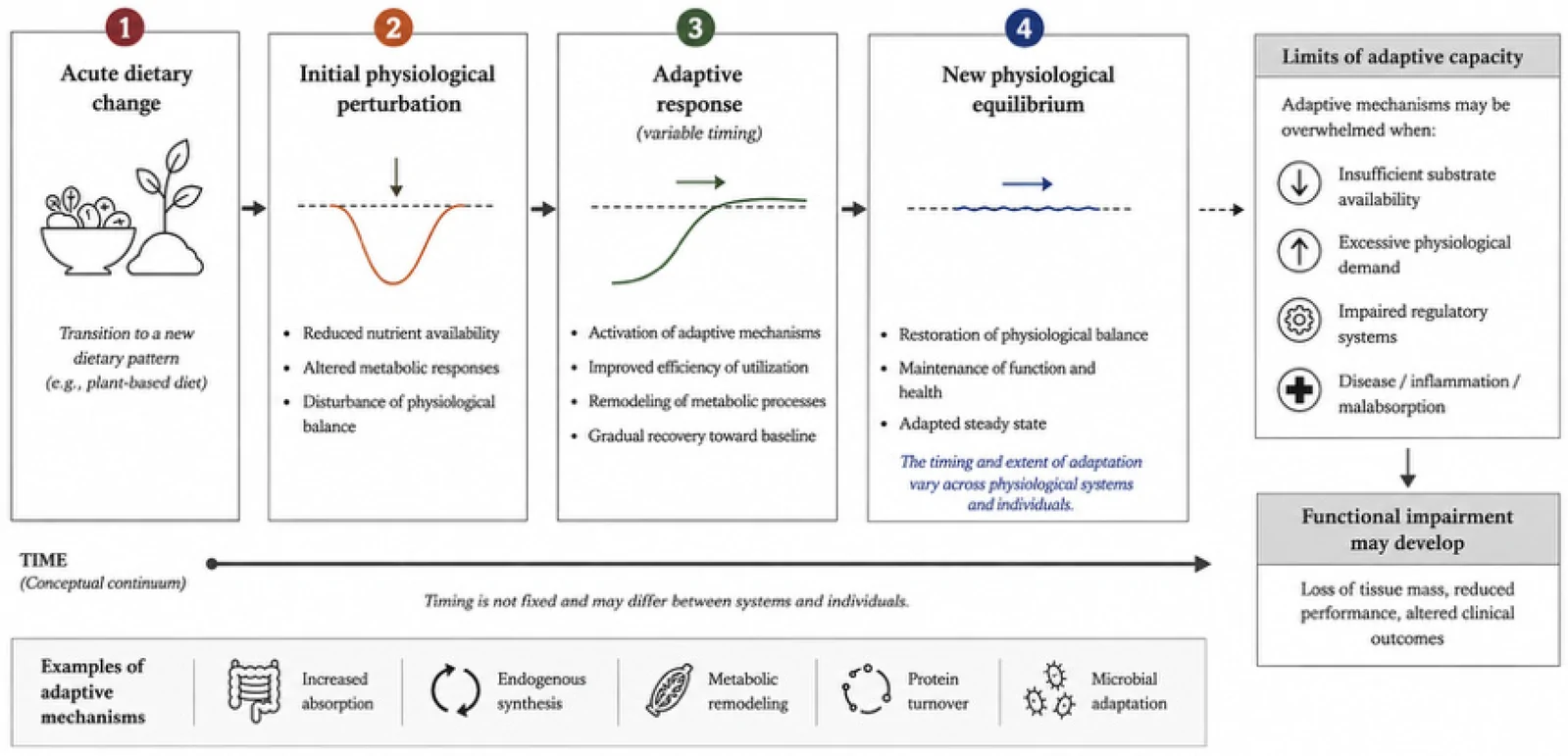
Conceptual model of physiological adaptation to sustained dietary change.

Living organisms maintain internal stability despite continuous fluctuations in nutrient availability through a complex network of homeostatic regulatory mechanisms. Rather than responding passively to dietary exposure, physiological systems dynamically adjust nutrient absorption, endogenous synthesis, metabolic utilization, storage, and excretion in order to preserve biological function [16,17,18,19]. Consequently, the relationship between dietary intake and physiological outcomes is not static but is continuously modulated by adaptive responses that buffer changes in nutrient availability and contribute to maintaining metabolic homeostasis (Figure 1).

### 2.1. Iron Homeostasis

Iron homeostasis represents one of the most illustrative examples of the complex relationship between dietary exposure and physiological function. Plant-based dietary patterns pose a distinctive nutritional challenge because they rely almost exclusively on non-heme iron, whereas omnivorous diets also provide heme iron, which is generally absorbed more efficiently. Furthermore, plant foods naturally contain compounds such as phytates and certain polyphenols that can reduce the acute bioavailability of non-heme iron by forming insoluble complexes within the intestinal lumen [16,17].

These characteristics have led iron to be regarded as one of the principal nutrients of concern in vegetarian and vegan diets, with discussions largely focusing on the lower bioavailability of dietary iron and the consequent risk of iron deficiency [18,19]. Accordingly, nutritional recommendations have primarily emphasized strategies aimed at increasing dietary iron availability, including higher recommended iron intakes, dietary planning to enhance iron absorption, food fortification, and supplementation in individuals at increased risk of deficiency [20,21]. However, these approaches are largely based on the assumption that lower dietary iron bioavailability necessarily translates into lower physiological iron availability. Whether this assumption adequately reflects iron homeostasis during long-term adherence to plant-based diets remains an important physiological question.

It is also worth distinguishing bioavailability from total intake: several dietary surveys report that mean total iron intake is as high as, or higher than, that of omnivores among vegetarians and vegans, since many staple plant foods—legumes, whole grains, leafy greens, and fortified cereals—are iron-dense. Lower fractional absorption of non-heme iron therefore does not necessarily translate into lower total iron delivered to the gut for absorption, which may partly explain why differences in iron status between dietary patterns are often smaller than differences in absorption efficiency alone would predict [22].

#### 2.1.1. Homeostatic Adaptive Mechanisms

Iron homeostasis is maintained through a tightly coordinated network of endocrine and cellular mechanisms that continuously adjust intestinal iron absorption according to systemic iron requirements rather than dietary iron intake alone [17]. Unlike many nutrients, iron balance is achieved almost exclusively by regulating absorption, since physiological iron losses are minimal and cannot be actively increased. Consequently, alterations in dietary iron availability are primarily counteracted by changes in intestinal iron transport rather than by modifications in iron excretion [16,23].

The hepatic peptide hormone hepcidin acts as the master regulator of systemic iron homeostasis. Adaptive regulation of hepcidin constitutes the principal mechanism through which the organism adjusts intestinal iron absorption to sustained changes in iron availability. When iron availability is reduced or iron demand increases, circulating hepcidin concentrations decrease, allowing for greater expression of the iron exporter ferroportin on enterocytes and macrophages [16,24]. This promotes the release of absorbed and recycled iron into the circulation, thereby increasing systemic iron availability. Conversely, elevated hepcidin concentrations induce ferroportin internalization and degradation, limiting intestinal iron absorption and iron recycling [16,25].

At the intestinal level, iron absorption is further regulated through coordinated changes in the expression and activity of key transport proteins. Ferric iron is initially reduced to its ferrous form by duodenal cytochrome b (Dcytb) at the apical membrane of enterocytes, after which it is transported into the cell via the divalent metal transporter 1 (DMT1) [26]. Once inside the enterocyte, iron can either be stored as ferritin or exported across the basolateral membrane through ferroportin, the only known cellular iron exporter [23,27]. The expression and activity of these transporters are dynamically regulated according to systemic iron requirements, enabling the intestine to adjust iron uptake efficiency in response to sustained changes in dietary iron availability.

Collectively, these regulatory mechanisms provide the physiological basis by which the organism maintains iron homeostasis despite substantial differences in dietary iron bioavailability. Rather than functioning as a passive consequence of dietary composition, intestinal iron absorption is a highly adaptive process that continuously responds to the body’s iron requirements. The extent to which these mechanisms translate into physiological adaptation during long-term adherence to plant-based dietary patterns has been explored in a growing number of human studies.

#### 2.1.2. Evidence for Long-Term Adaptation

The physiological mechanisms regulating iron absorption are well-established. A key question, however, is whether these regulatory responses translate into measurable adaptations during the long-term consumption of diets characterized by lower iron bioavailability. Over the past two decades, accumulating human evidence has progressively shown that intestinal iron absorption adapts according to habitual dietary exposure rather than remaining fixed.

The first experimental evidence was provided by Hunt and Roughead, who demonstrated that healthy adults consuming diets with lower iron bioavailability for several weeks exhibited significantly greater fractional non-heme iron absorption than those consuming diets with higher iron bioavailability, despite similar iron intakes [28]. These results challenged the assumption that acute estimates of iron bioavailability necessarily predict long-term iron absorption. This concept was subsequently reinforced by Armah et al., who reported that women consuming a high-phytate diet for eight weeks showed significantly greater fractional iron absorption than before the intervention, despite continued exposure to phytate [29]. These findings contrast with single meal studies in which phytate exposure can significantly impair iron absorption [30,31]. Therefore, these longer-term studies demonstrate that intestinal iron absorption is not a fixed physiological characteristic but a plastic process that responds to habitual dietary exposure, which we cannot infer from acute feeding studies.

Further support has come from observational studies. Ambroszkiewicz et al. found that vegetarian children exhibited lower circulating hepcidin concentrations than omnivorous children while maintaining normal iron status, consistent with enhanced physiological regulation of iron absorption in response to habitual dietary intake [32]. More recently, a controlled trial comparing long-term vegans and omnivores demonstrated a significantly greater postprandial absorption of non-heme iron in individuals habitually consuming a vegan diet following the ingestion of an identical standardized test meal. This enhanced absorptive response was accompanied by lower circulating hepcidin concentrations in the vegan group (13.6 ± 8.0 vs. 24.3 ± 9.0 ng/mL in omnivores; *p* = 0.02; probability of superiority [PS] = 0.58), together with a greater serum iron incremental area under the curve (iAUC; 1002.8 ± 143.9 vs. 853.9 ± 268.2 µmol/L/h; *p* = 0.04; effect size [ES] = 0.68). These findings provide physiological evidence consistent with the adaptive regulation of iron homeostasis in individuals habitually consuming a vegan diet. Although the standardized test-meal approach was appropriate for assessing differences in postprandial iron handling associated with habitual dietary exposure, further longitudinal studies and research in populations with specific physiological or clinical characteristics are needed to further characterize the extent and limits of this adaptive response [33].

These findings collectively challenge the widespread assumption that acute estimates of dietary iron bioavailability necessarily predict long-term iron status. Instead, they support a more dynamic model in which physiological adaptation actively modulates the relationship between dietary exposure and systemic iron homeostasis.

#### 2.1.3. When Adaptation Becomes Insufficient

The available evidence indicates that in healthy individuals, adaptive regulation of iron absorption is generally sufficient to preserve normal erythropoiesis despite the lower bioavailability of dietary iron [34]. Consistent with this concept, comparative studies generally report no clinically meaningful differences in hemoglobin concentrations between vegetarians, vegans, and omnivores, suggesting that adaptive increases in iron absorption are often sufficient to preserve erythropoiesis under normal physiological conditions [35,36,37,38]. However, hemoglobin is a relatively late indicator of iron deficiency and does not necessarily reflect body iron stores. Accordingly, the maintenance of normal hemoglobin concentrations should not be interpreted as evidence that adaptive capacity is unlimited.

Rather, the capacity to increase intestinal iron absorption operates within physiological boundaries and may become insufficient when iron requirements exceed absorptive capacity or when additional factors disrupt iron homeostasis [23]. Physiological conditions associated with increased iron requirements represent the clearest examples of these limitations. During pregnancy, maternal iron demands increase substantially to support fetal growth, placental development, and expansion of maternal blood volume, while regular menstrual blood losses and periods of rapid growth, such as adolescence, also increase the iron requirements [39,40]. Under these circumstances, adaptive increases in intestinal iron absorption may be insufficient to fully compensate for increased physiological demands, particularly when dietary iron intake is suboptimal.

The effectiveness of adaptive mechanisms may also be impaired by pathological conditions that interfere with iron regulation or intestinal iron absorption. Chronic inflammation stimulates hepcidin production, thereby reducing ferroportin-mediated iron export and limiting intestinal iron absorption irrespective of dietary iron intake [41,42]. As a consequence, inflammation can also lead to elevated ferritin concentrations, which may partly explain the higher ferritin levels observed in omnivores than in vegans in some cohorts, without necessarily reflecting a better iron status [34]. Finally, gastrointestinal disorders that compromise intestinal integrity or nutrient absorption, including celiac disease, inflammatory bowel disease, and conditions following gastrointestinal surgery, may reduce the capacity to adapt to diets characterized by lower iron bioavailability [43,44].

These observations reinforce an important physiological concept: adaptation reduces, but does not eliminate, the nutritional challenges associated with lower dietary iron bioavailability. Consequently, dietary counseling, food fortification, or iron supplementation may still be required in individuals with increased physiological requirements, impaired iron regulation, or multiple concurrent risk factors for iron deficiency [40]. Rather than viewing lower dietary iron bioavailability as inevitably leading to iron deficiency, or physiological adaptation as providing complete protection, current evidence supports a more nuanced interpretation in which iron status reflects the balance between dietary exposure, homeostatic adaptation, and individual physiological requirements.

### 2.2. Endogenous Biosynthesis as a Physiological Adaptation

Unlike essential nutrients, several bioactive compounds found predominantly in animal-source foods—including creatine and carnosine—can also be synthesized endogenously from dietary amino acid precursors. Nevertheless, plant-based dietary patterns provide little or no preformed amounts of these compounds, resulting in substantially lower dietary exposure than omnivorous diets [45,46]. Consequently, individuals adhering to vegetarian and vegan dietary patterns have frequently been assumed to be at increased risk of functional insufficiency owing to the absence of preformed dietary sources, potentially contributing to impaired muscular, neurological, or metabolic function [47].

This interpretation, however, implicitly assumes that tissue concentrations depend primarily on dietary intake. In reality, circulating and tissue levels of these compounds reflect the balance between dietary exposure, endogenous biosynthesis, tissue uptake, metabolic utilization, and excretion. Therefore, reduced dietary intake does not necessarily imply proportional reductions in biological availability.

#### 2.2.1. Homeostatic Adaptive Mechanisms

The principal adaptive response to reduced dietary exposure is the upregulation of endogenous biosynthesis. Creatine is synthesized primarily in the kidneys and liver through sequential reactions involving arginine:glycine amidinotransferase (AGAT) and guanidinoacetate methyltransferase (GAMT), with synthesis being tightly regulated according to tissue creatine availability [48,49]. Likewise, carnosine is synthesized endogenously from β-alanine and L-histidine, predominantly in skeletal muscle and nervous tissue, where it contributes to intracellular buffering and other physiological functions [50].

Beyond increased biosynthesis, homeostasis may also be maintained through adaptations affecting tissue transport, intracellular conservation, and metabolic utilization. Although these mechanisms have been less extensively investigated in humans than endogenous synthesis itself, they contribute to maintaining tissue availability despite prolonged reductions in dietary intake. Consequently, tissue concentrations are determined by the integrated regulation of synthesis, transport, utilization, and turnover rather than dietary exposure alone.

#### 2.2.2. Evidence for Long-Term Adaptation

Human studies consistently demonstrate that individuals adhering to vegetarian and vegan dietary patterns consume negligible or substantially lower amounts of preformed creatine and carnosine than omnivores [51]. However, unlike essential nutrients, these compounds can be synthesized endogenously from amino acid precursors and are therefore not considered dietary essentials under normal physiological conditions [52]. Consequently, lower dietary exposure does not necessarily translate into inadequate biological availability but rather modifies the relative contribution of endogenous biosynthesis and dietary intake to tissue homeostasis.

Among these compounds, creatine provides the clearest evidence supporting this adaptive concept. Vegetarians generally exhibit lower intramuscular creatine concentrations than omnivores, reflecting the absence of dietary creatine intake rather than impaired creatine homeostasis [53,54,55]. Importantly, these lower baseline concentrations are typically compatible with normal physiological function, indicating that endogenous creatine synthesis is generally sufficient to meet habitual metabolic requirements in healthy individuals [52].

Evidence from supplementation studies provides further insight into this adaptive response. Compared with omnivores, vegetarians consistently exhibit greater increases in intramuscular total creatine and phosphocreatine concentrations following creatine supplementation, frequently accompanied by larger improvements in lean body mass and high-intensity exercise performance [54,55,56]. Rather than indicating a pre-existing physiological deficiency, this enhanced responsiveness is more plausibly explained by lower baseline tissue saturation resulting from chronically reduced dietary exposure [57]. From this perspective, these findings suggest that endogenous creatine synthesis is generally sufficient to preserve physiological function under habitual conditions, whereas exogenous creatine may confer additional physiological benefits when long-term dietary exposure has resulted in lower baseline tissue saturation.

Although considerably fewer human data are available for carnosine, circulating concentrations are generally maintained within physiological ranges in healthy vegetarians and vegans despite negligible dietary intake [58,59]. While further research is required to characterize tissue-specific adaptations, the available evidence suggests that endogenous biosynthesis similarly contributes to maintaining the homeostasis of these metabolites under conditions of chronically reduced dietary exposure [60].

#### 2.2.3. When Adaptation Becomes Insufficient

As with other homeostatic systems, endogenous biosynthesis has physiological limits. Endogenous production appears to be regulated to preserve physiological homeostasis rather than to maximize tissue concentrations or functional reserve. Consequently, conditions associated with increased metabolic demands may exceed the capacity of endogenous synthesis alone and increase the physiological relevance of dietary intake or supplementation [49,61].

This concept is particularly relevant for creatine. Although endogenous synthesis appears sufficient to maintain normal physiological function in healthy individuals, accumulating evidence suggests that exogenous creatine supplementation may provide additional benefits in situations characterized by increased energetic demands, including high-intensity athletic performance, aging-associated sarcopenia, neurodegenerative disorders, and certain neuromuscular diseases [56,62]. Importantly, these observations should not be interpreted as evidence that endogenous synthesis is inadequate under normal conditions, but rather that physiological requirements may extend beyond those necessary to maintain basal homeostasis.

The adaptive capacity of endogenous biosynthesis may also be influenced by the availability of precursor amino acids and the integrity of the enzymatic pathways involved. Conditions affecting hepatic or renal function, inherited defects in creatine biosynthesis, severe protein-energy malnutrition, or disorders associated with altered sulfur amino acid metabolism may compromise the synthesis of creatine or carnosine, thereby increasing dependence on dietary intake or supplementation [49].

### 2.3. Protein

Protein metabolism differs fundamentally from that of many other nutrients because amino acids continuously participate in dynamic processes of protein synthesis, degradation, oxidation, and recycling rather than existing as static body stores [63,64]. Consequently, maintaining protein homeostasis depends not only on dietary protein intake, but also on the coordinated regulation of whole-body protein turnover and nitrogen metabolism [65].

Plant-based dietary patterns present a distinctive physiological challenge because although the total protein intake frequently meets the current recommendations established by the Institute of Medicine (IOM) and the European Food Safety Authority (EFSA) [66,67], the protein supplied generally exhibits lower digestibility, and depending on dietary composition, lower proportions of one or more indispensable amino acids than diets containing animal-source foods [68,69]. For this reason, plant-based diets have traditionally been regarded as potentially suboptimal for supporting muscle protein metabolism, particularly in populations with increased physiological requirements [70]. Accordingly, nutritional strategies have largely focused on increasing total protein intake, combining complementary plant protein sources, improving protein quality, or recommending protein supplementation to compensate for differences in digestibility and amino acid composition [71,72].

Collectively, these approaches are based on the implicit assumption that lower dietary protein quality and digestible indispensable amino acid supply translate directly into proportional reductions in physiological protein availability. Whether this assumption adequately reflects the regulation of protein homeostasis during long-term adherence to plant-based dietary patterns remains an important physiological question.

#### 2.3.1. Homeostatic Adaptive Mechanisms

Protein homeostasis is maintained through several complementary adaptive mechanisms that improve the efficiency of amino acid utilization during sustained reductions in dietary protein availability, thereby preserving whole-body protein homeostasis across a broad range of physiologically adequate protein intakes [73,74]. One of the best-characterized adaptive responses is an improvement in nitrogen economy. Sustained reductions in habitual protein intake are accompanied by progressive decreases in amino acid oxidation and urea production, thereby conserving indispensable amino acids for protein synthesis rather than irreversible catabolism [75,76]. These metabolic adjustments increase the efficiency with which dietary amino acids are utilized and contribute to the maintenance of nitrogen balance despite lower protein availability [77]. Protein turnover is likewise adaptively regulated through coordinated adjustments in protein synthesis and degradation according to dietary protein availability, energy status, endocrine regulation, and physiological demand. By continuously remodeling these processes, the organism preserves essential protein functions while minimizing unnecessary amino acid losses during sustained dietary change [74,78].

A further component of this adaptive response is the efficient recycling of endogenous amino acids. Because most amino acids released during protein degradation are reutilized for subsequent rounds of protein synthesis, only a relatively small proportion is irreversibly oxidized or lost each day. This metabolic efficiency reduces the need for the continuous replacement of amino acids from the diet and contributes to maintaining protein homeostasis during sustained fluctuations in dietary protein availability [64,79].

Together, these mechanisms illustrate that physiological protein availability cannot be inferred solely from dietary protein quantity or quality. Instead, whole-body protein homeostasis emerges from the dynamic interaction between dietary amino acid supply and the adaptive regulation of protein metabolism.

#### 2.3.2. Evidence for Long-Term Adaptation

Evidence from human studies indicates that well-planned plant-based dietary patterns providing adequate total protein intake generally preserve muscle mass and functional outcomes during long-term interventions [11,80]. Rather than supporting the assumption that lower dietary protein digestibility inevitably compromises physiological function, the available evidence suggests that whole-body protein homeostasis can be maintained despite differences in dietary protein quality, consistent with the adaptive mechanisms described above [68,69].

Initial concerns regarding plant-based proteins largely originated from acute metabolic studies evaluating postprandial muscle protein synthesis following the ingestion of isolated protein sources. Several early investigations reported lower anabolic responses after the ingestion of certain plant-derived proteins compared with rapidly digested animal proteins such as whey [81,82,83]. However, subsequent studies demonstrated that these differences become substantially attenuated, and in some experimental settings disappear altogether, when the total protein intake is sufficient, complementary protein sources are consumed, or adequate leucine intake is achieved [83,84,85,86,87]. Moreover, recent controlled feeding studies using integrated measurements of daily myofibrillar or mixed muscle protein synthesis have reported comparable rates between omnivorous and exclusively plant-based dietary patterns over 9–10 days [88,89]. In further support of these findings, a quasi-experimental study found that meals containing foods with an “incomplete” essential amino acid profile stimulated comparable 24-h muscle protein synthesis to meals with complete or complementary essential amino acid profiles [90]. These findings highlight the complexity of the relationship between protein intake and muscle protein synthesis [91].

Evidence from longer-term randomized controlled trials provides a more appropriate framework for determining whether differences observed at the level of protein intake and muscle protein synthesis translate into meaningful changes in physiological outcomes, because these studies capture the cumulative effects of sustained dietary exposure rather than isolated metabolic responses. Across these interventions, plant-based dietary patterns providing adequate protein intake consistently preserve lean body mass, skeletal muscle mass, and muscular strength to a similar extent as omnivorous diets [69]. Consistent with these findings, a recent systematic review and meta-analysis of randomized controlled trials found no significant differences between plant-based and omnivorous dietary patterns for upper-body strength, lower-body strength, or overall muscular strength [92]. Taken as a whole, these findings indicate that differences in dietary protein quality are not sufficient, by themselves, to predict long-term physiological function. Rather, the preservation of functional outcomes is consistent with the capacity of whole-body protein metabolism to adapt to differences in dietary protein characteristics, although these functional outcomes do not directly demonstrate the underlying adaptive mechanisms.

#### 2.3.3. When Adaptation Becomes Insufficient

Although the adaptive regulation of protein metabolism is highly effective under normal physiological conditions, its capacity is not unlimited. Adaptive capacity ultimately depends on the interaction between three interrelated factors: substrate availability, metabolic demand, and the integrity of the regulatory systems responsible for maintaining protein homeostasis. Consequently, when dietary protein availability becomes insufficient, metabolic demand increases substantially, or the physiological mechanisms regulating protein metabolism are impaired, adaptive responses may no longer be sufficient to preserve protein homeostasis, increasing the physiological importance of dietary protein quantity and quality [74,76].

One important circumstance in which adaptive capacity may become insufficient is prolonged exposure to protein intakes below physiologically adequate levels. Although homeostatic mechanisms improve nitrogen economy and amino acid utilization, they cannot fully compensate when dietary protein availability falls below the minimum required to sustain tissue protein synthesis. Under these conditions, persistent negative nitrogen balance, progressive reductions in body cell mass, and impaired muscle function may ultimately develop despite ongoing metabolic adaptation [93]. This situation remains particularly relevant in populations experiencing chronic food insecurity or limited access to adequate protein-rich foods, where insufficient protein intake reflects constrained dietary availability rather than dietary choice [91].

Adaptive capacity may also be compromised in conditions characterized by profound disturbances of protein metabolism, including severe systemic inflammation, major trauma, extensive burns, advanced chronic disease, or gastrointestinal disorders associated with protein malabsorption [94,95,96]. Under these circumstances, accelerated protein turnover, increased amino acid oxidation, impaired nutrient absorption, or reduced anabolic responsiveness may overwhelm the capacity of homeostatic mechanisms to maintain protein balance, thereby increasing dependence on adequate dietary protein intake, and when appropriate, nutritional support.

### 2.4. Fermentable Carbohydrates

Unlike most nutrients absorbed in the small intestine, a substantial proportion of dietary fiber, resistant starch, galacto-oligosaccharides (GOSs), and other fermentable carbohydrates escape host digestion and reach the colon largely intact, where they become substrates for microbial fermentation rather than direct human metabolism [97,98]. Consequently, the physiological effects of these dietary components depend not only on their chemical structure or quantity, but also on the metabolic capacity of the gut microbiota to utilize them [99].

Plant-based dietary patterns are typically characterized by substantially greater intakes of dietary fiber and other fermentable substrates than omnivorous diets, including resistant starch, GOS, fructans, and diverse non-digestible polysaccharides [100,101]. This abrupt increase in substrate availability alters the ecological and metabolic environment of the colon, frequently resulting in increased microbial fermentation and transient elevations in hydrogen, carbon dioxide, methane, and short-chain fatty acid production [102,103,104,105]. During the early stages of dietary transition, these changes may be accompanied by gastrointestinal symptoms such as flatulence, bloating, or abdominal discomfort, which are commonly perceived as evidence of poor tolerance to plant-based foods.

Consequently, efforts to improve gastrointestinal tolerance have traditionally focused on strategies such as the gradual introduction of fiber-rich foods, food processing techniques that reduce the fermentable carbohydrate content of foods, enzyme supplementation, or, in susceptible individuals, the temporary restriction of specific fermentable carbohydrates [106,107,108]. Although these approaches may be appropriate for managing symptoms in selected clinical contexts, they place comparatively less emphasis on the capacity of the gut microbiota to adapt to sustained increases in fermentable substrate availability. Whether these early gastrointestinal responses reflect persistent intolerance or a transient phase preceding functional adaptation of the gut microbial ecosystem remains an important physiological question.

#### 2.4.1. Homeostatic Adaptive Mechanisms

The adaptive response to sustained increases in fermentable substrate availability is primarily mediated through functional remodeling of the gut microbiota. Rather than representing a static microbial community, the intestinal microbiota continuously adjusts its composition and metabolic activity according to the availability of fermentable substrates reaching the colon [100,109]. One of the principal adaptive responses is the selective enrichment of microbial taxa capable of degrading complex carbohydrates. Increased exposure to dietary fiber, resistant starch, galacto-oligosaccharides, and other non-digestible carbohydrates promotes the expansion of saccharolytic microorganisms possessing carbohydrate-active enzymes (CAZymes), thereby increasing the efficiency with which these substrates are metabolized [110,111]. As microbial communities adapt to habitual substrate availability, their collective fermentative capacity progressively increases.

Microbial adaptation is further reinforced through metabolic cross-feeding, whereby fermentation products generated by primary degraders serve as substrates for secondary microbial species [112,113]. These cooperative metabolic interactions enhance ecosystem stability, increase substrate utilization efficiency, and promote the production of short-chain fatty acids (SCFAs), particularly acetate, propionate, and butyrate, which contribute to colonic epithelial health, intestinal barrier integrity, and host metabolic regulation [114,115].

#### 2.4.2. Evidence for Long-Term Adaptation

Longitudinal human intervention studies provide the most appropriate framework for evaluating physiological adaptation to fermentable carbohydrates because they capture the cumulative effects of sustained dietary exposure rather than the transient responses observed immediately after dietary change. Across controlled dietary interventions involving legumes and other fermentable substrates, gastrointestinal symptoms such as flatulence, bloating, and abdominal discomfort generally decline over time despite continued dietary exposure [106,116,117,118]. Interestingly, improvements in gastrointestinal tolerance are not always accompanied by proportional reductions in intestinal gas production. Early controlled feeding studies demonstrated that repeated consumption of legumes progressively reduced the perception of flatulence and digestive discomfort despite relatively small changes in the total volume of intestinal gas produced [105,116]. More recent intervention studies have similarly reported that regular consumption of legumes or galacto-oligosaccharides is generally well-tolerated following an initial adaptation period, supporting the concept that clinical adaptation reflects more than a simple reduction in microbial gas production [104,117,118].

The reproducibility of these observations across different fermentable substrates, intervention protocols, and study populations supports the concept that gastrointestinal tolerance is a dynamic physiological characteristic that evolves with sustained dietary exposure rather than a fixed response determined by the initial digestive experience [104,106,118]. This raises an important unresolved question: whether the pre-existing state of the gut microbiota may influence the magnitude or trajectory of this adaptive response. Antibiotic exposure and dysbiosis can substantially perturb the gut microbial ecosystem, but whether such perturbations identify individuals with a reduced capacity to adapt to increased fermentable carbohydrate intake remains unknown [119,120]. Prospective studies integrating baseline microbiota characteristics with longitudinal measures of gastrointestinal tolerance and microbial function could help determine whether specific features of the pre-existing gut ecosystem predict individual differences in adaptation and could ultimately serve as clinically useful biomarkers.

Taken together, these findings indicate that the digestive symptoms frequently experienced during the transition to plant-based dietary patterns should be interpreted as part of a dynamic adaptive process. Acute physiological responses following dietary change do not necessarily predict the functional equilibrium achieved after long-term adaptation.

#### 2.4.3. When Adaptation Becomes Insufficient

Adaptive capacity ultimately depends on three interrelated factors: sustained exposure to fermentable substrates, the resilience and functional diversity of the gut microbial community, and the integrity of the gastrointestinal environment [121]. Consequently, conditions that impair microbial diversity or disrupt host–microbiota interactions may reduce the capacity to adapt to increased intakes of fermentable carbohydrates [120,122,123].

Adaptive capacity may be compromised in gastrointestinal disorders characterized by altered microbial composition or impaired intestinal function, including irritable bowel syndrome (IBS), inflammatory bowel disease (IBD), and other conditions associated with intestinal dysbiosis [119,124]. In these individuals, increased fermentation of dietary fiber or other fermentable carbohydrates may provoke persistent gastrointestinal symptoms despite repeated exposure, and temporary dietary modifications, including low-FODMAP approaches, may be clinically appropriate during symptom management [125,126]. However, these interventions should not be interpreted as evidence that fermentable carbohydrates are intrinsically poorly tolerated, but rather that the adaptive capacity of the gut ecosystem may be impaired under specific pathological conditions.

Adaptive capacity may also be transiently impaired following major perturbations of the gut microbial ecosystem, including recent antibiotic exposure or gastrointestinal infections, which may delay the establishment of microbial adaptation [120,127]. Consequently, the physiological consequences of increasing fermentable carbohydrate intake depend not only on the amount of fermentable substrate reaching the colon, but also on the capacity of the gut microbial ecosystem to establish and maintain functional adaptation.

### 2.5. Plant Polyphenols

Plant-based dietary patterns are naturally rich in polyphenols, including hydroxybenzoic acids, hydroxycinnamic acids, flavonoids, lignans, and condensed tannins [128]. However, the biological effects of many dietary polyphenols depend not only on their dietary intake, but also on their transformation by the gut microbiota into smaller, more readily absorbable and biologically active metabolites. Many polyphenols occur in foods in complex or conjugated forms, limiting their direct absorption in the gastrointestinal tract. Consequently, the biological availability of dietary polyphenols cannot be inferred solely from their intake or their chemical abundance in foods [129]. Rather, it depends on the capacity of the gut microbial ecosystem to metabolize these compounds and on the host’s subsequent absorption and metabolism of the resulting products [130]. In this context, sustained exposure to a polyphenol-rich plant-based diet may influence gut microbial metabolism and the biotransformation of dietary polyphenols into bioactive metabolites.

#### 2.5.1. Homeostatic Adaptive Mechanisms

The gut microbiota is a dynamic ecosystem capable of metabolizing a wide range of dietary polyphenols, with specific microbial taxa contributing to the conversion of these compounds into smaller metabolites. These include *Faecalibacterium prausnitzii*, *Bifidobacterium longum*, *Bifidobacterium pseudocatenulatum*, Oscillibacter, and Roseburia species [131,132,133]. Similarly, replacement of animal protein sources with legumes has been associated with increased abundance of *Eubacterium rectale*, *Roseburia faecis*, *Roseburia hominis*, and Bifidobacterium species [134]. These microorganisms have been implicated in the biotransformation of dietary polyphenols into smaller metabolites that can be absorbed and further metabolized by the host.

Specifically, *Faecalibacterium prausnitzii* is positively associated with fecal and urinary concentrations of enterolactone [135,136], a lignan-derived metabolite. This association appears to be strengthened by higher fiber intake, suggesting that the fiber-associated microbial environment may facilitate lignan biotransformation [135]. Consumption of whole grains, particularly oats, has also been associated with an increased abundance of *F. prausnitzii* and greater biotransformation of avenanthamides into dihydro-AVA metabolites [137]. Furthermore, *F. prausnitzii*, together with Eubacterium and Oscillibacter, is positively associated with hippuric acid concentrations, a metabolite derived largely from the microbial catabolism of catechins and chlorogenic acids present in plant foods [138,139]. Consistent with these observations, individuals following plant-based diets exhibit higher concentrations of several chlorogenic acid-derived metabolites, including hippuric acid [128,129,140].

Eubacterium and Oscillibacter are also positively associated with urinary equol concentrations, reflecting the microbial conversion of daidzein from soyfoods [141]. In the Health Study, individuals following vegan diets excreted 4.4-fold and 3-fold higher concentrations of urinary enterolactone and equol, respectively, than non-vegetarian individuals [142]. Similarly, Setchell et al. found that the proportion of equol producers was higher among vegetarians (59%) than among non-vegetarians, with vegetarians being 4.25 times more likely to be equol producers [143].

Specific plant foods provide further examples of microbiota-dependent polyphenol biotransformation. *Bifidobacterium pseudocatenulatum* and *Bifidobacterium longum*, owing to their myrosinase-like activity, can contribute to the conversion of glucoraphanin and glucotropaeolin from cruciferous vegetables into sulforaphane [144,145]. Similarly, the consumption of nuts, particularly walnuts, has been associated with an increased abundance of Gordonibacter, a genus involved in the biotransformation of ellagitannins into urolithin A [146].

Together, these examples illustrate the central role of the gut microbiota in determining the metabolic fate of plant-derived polyphenols and the resulting exposure to bioactive metabolites.

#### 2.5.2. Evidence for Long-Term Adaptation

Evidence from intervention studies provides a stronger basis for considering whether sustained polyphenol exposure can modify the gut microbial metabolism. Controlled dietary interventions have shown that the regular consumption of polyphenol-rich foods can alter gut microbial composition. For example, a polyphenol-rich dietary intervention over 8 weeks increased fiber-fermenting and butyrate-producing bacteria, including members of the Ruminococcaceae family and the genus Faecalibacterium [147]. Similarly, a controlled intervention with red wine polyphenols increased several bacterial groups, including Bifidobacterium, Bacteroides, and Prevotella [148].

These intervention findings complement observational evidence linking habitual plant-based dietary patterns with differences in polyphenol-derived metabolites. Greater adherence to a healthy plant-based diet has been associated with higher plasma concentrations of polyphenol-derived metabolites, including trigonelline, O-methylcatechol sulfate, catechol sulfate, and hippuric acid [128,140]. Similarly, individuals following vegan diets exhibit higher plasma concentrations of benzoate-derived metabolites and higher urinary concentrations of enterolactone and equol than non-vegetarian individuals [142,143]. These findings indicate that sustained exposure to plant-based dietary patterns is associated with measurable differences in the systemic and urinary metabolite profiles derived from plant foods. However, whether these changes reflect functional adaptation of microbial polyphenol metabolism, rather than simply greater substrate availability, remains uncertain. Demonstrating functional adaptation would require evidence that sustained exposure alters the microbial capacity to biotransform polyphenols beyond the increase expected from greater substrate availability alone.

#### 2.5.3. When Adaptation Becomes Insufficient

The capacity of the gut microbiota to biotransform dietary polyphenols is not uniform across individuals and may be influenced by microbial composition, habitual dietary exposure, gastrointestinal health, and other host-related factors. The marked interindividual variability in the production of metabolites such as equol illustrates that exposure to the same dietary substrate does not necessarily result in the same metabolic response [149,150]. Conditions that substantially disrupt the gut microbial ecosystem or impair intestinal function may therefore alter the capacity to generate bioactive metabolites from plant-derived compounds [151,152,153]. Consequently, dietary polyphenol exposure should not be interpreted solely from food intake, but in relation to the functional capacity of the gut microbiota and the host to metabolize these compounds.

## 3. Integrating Physiological Adaptation into Nutritional Science

The evidence reviewed throughout this article supports a broader conceptual framework for interpreting nutritional physiology. Across multiple biological systems—including iron homeostasis, endogenous biosynthesis, protein metabolism, and the gut microbial ecosystem—a common physiological principle emerges: the biological consequences of dietary exposure are determined not only by nutrient intake but also by the homeostatic adaptive mechanisms that regulate physiological function during sustained dietary exposure [28,53,74,105] (Table 1). Consequently, dietary composition should be viewed as the starting point of physiological regulation rather than its sole determinant.

An important implication of this framework is the need to distinguish between acute physiological responses and the homeostatic equilibrium established after long-term adaptation. Although acute metabolic studies provide valuable mechanistic insights, they do not necessarily predict the functional state ultimately achieved following sustained dietary exposure [28,105,154]. Failure to distinguish between these temporal dimensions may lead to the overinterpretation of acute physiological responses while underestimating the capacity of adaptive mechanisms to maintain long-term homeostasis.

Recognizing physiological adaptation does not imply that nutrient composition is unimportant or that all dietary patterns are physiologically equivalent. Rather, it emphasizes that dietary recommendations should consider both nutrient exposure and the capacity of physiological regulatory systems to maintain homeostasis. This distinction becomes particularly relevant in situations where adaptive capacity may be exceeded because of increased physiological demands or pathological conditions, in which dietary interventions or supplementation remain essential.

Overall, incorporating physiological adaptation into nutritional science provides a more comprehensive framework for understanding how dietary patterns influence human health. Ultimately, nutrient intake represents the input to human physiology, whereas long-term physiological function reflects the integrated output of adaptive homeostatic regulation.

From a clinical and dietetic perspective, this framework has a direct practical implication: counseling around plant-based diets should extend beyond static nutrient-of-concern checklists to also consider adaptation timelines and individual adaptive capacity. Patients newly transitioning to a plant-based diet, or those in physiological states associated with reduced regulatory capacity—including pregnancy, active inflammatory or gastrointestinal disease, and recovery from disordered eating— may benefit from closer nutritional monitoring during the adaptation period, while patients with long, stable adherence and no complicating conditions may require less intensive intervention than nutrient-of-concern checklists alone would suggest.

## 4. Future Research Priorities

Despite growing evidence supporting physiological adaptation across multiple biological systems, several important questions remain unresolved. Addressing these knowledge gaps will be essential for improving our understanding of how adaptive processes influence the long-term physiological consequences of dietary patterns and for integrating this perspective into nutritional science.

One important area requiring further investigation concerns the temporal dynamics of physiological adaptation. Most dietary intervention studies evaluate outcomes after only a few weeks of dietary exposure, providing limited insight into when adaptive responses are initiated, how they evolve over time, or when a new homeostatic equilibrium is established. Longitudinal studies incorporating repeated measurements throughout the adaptation process, rather than relying solely on baseline and end-of-intervention assessments, would allow for the characterization of adaptation trajectories and provide a more comprehensive understanding of the transition from acute physiological responses to long-term homeostasis [155].

Another important challenge is the identification of biomarkers capable of distinguishing transient physiological responses from stable adaptive states. Current nutritional research relies predominantly on biomarkers that quantify nutrient status or metabolic function, yet relatively few indicators directly reflect the progression or completion of physiological adaptation. The development of such biomarkers could substantially improve the interpretation of dietary intervention studies by differentiating temporary metabolic responses from stable physiological regulation.

Future research should also seek to better understand the determinants of interindividual variability in adaptive capacity. The extent and rate of physiological adaptation are likely influenced by multiple factors, including age, genetic background, habitual dietary patterns, metabolic health, physical activity, and the composition and functional capacity of the gut microbiota [10,156]. Elucidating these sources of variability may contribute to more individualized nutritional recommendations and improve the identification of populations in whom adaptive mechanisms are either enhanced or compromised.

Chronic disease states that alter baseline regulatory capacity may be a particularly informative test case. For example, in a 12-week randomized controlled trial in adults with type 1 diabetes, a fiber-rich, plant-based dietary intervention was associated with reduced daily insulin requirements; a secondary analysis of this trial further found that higher dietary lignan intake—a marker of fiber and plant food intake—was associated with lower insulin requirements independent of change in body weight [157]. Whether this reflects an adaptive metabolic response of the kind described throughout this review or a distinct mechanism specific to impaired insulin-dependent glucose regulation remains to be clarified, but it illustrates how populations with altered baseline regulatory capacity could serve as a useful model for probing the limits of physiological adaptation to dietary change.

Finally, future nutritional research may benefit from adopting a more integrative physiological perspective. Rather than evaluating nutrients as isolated dietary exposures, intervention studies should increasingly characterize the coordinated adaptive responses that link dietary change with long-term physiological function. Such an approach would provide a more comprehensive understanding of how homeostatic regulation shapes the biological consequences of dietary exposure and may ultimately improve both the interpretation of nutritional evidence and the development of evidence-based dietary recommendations.

## 5. Conclusions

Plant-based dietary patterns provide a unique physiological model for understanding how the human organism adapts to sustained dietary change. As illustrated throughout this review, differences in nutrient composition, digestibility, or bioavailability do not necessarily translate into proportional differences in long-term physiological function because adaptive homeostatic mechanisms continuously regulate nutrient absorption, metabolism, synthesis, utilization, and microbial function. Recognizing these dynamic physiological responses provides a more comprehensive framework for interpreting the health effects of plant-based dietary patterns while acknowledging that adaptive capacity operates within defined biological limits and may be exceeded under specific physiological or pathological conditions.

More broadly, the concept of physiological adaptation extends beyond plant-based nutrition and has important implications for nutritional science as a whole. Rather than considering dietary intake as the sole determinant of physiological function, nutritional assessment should increasingly recognize that long-term biological outcomes emerge from the continuous interaction between dietary exposure and adaptive homeostatic regulation. Incorporating this perspective into nutritional research and dietary recommendations may improve the interpretation of dietary interventions and provide a more physiologically grounded framework for understanding the relationship between diet and human health.

## Figures and Tables

**Table 1:** From dietary exposure to physiological adaptation: common adaptive responses across biological systems.

System	Dietary Exposure	Initial Perturbation	Adaptive Response	Adapted Physiological Outcome	Limits of Adaptation
Iron	↓ bioavailable iron	↓ absorbed iron	↑ absorption	Iron balance maintained	Pregnancy, inflammation
Endogenous synthesis	↓ dietary creatine/carnosine	↓ exogenous supply	↑ synthesis	Tissue availability maintained	Enzyme defects, high demand
Protein	↓ digestible indispensable amino acids	↓ anabolic stimulus	Nitrogen conservation, recycling	Muscle preserved	Protein insufficiency, catabolic disease
Fermentable carbohydrates	↑ fiber/FODMAPs	↑ fermentation symptoms	Microbial remodeling	Improved tolerance	IBS, dysbiosis
Plant polyphenols	↑ polyphenol intake	Variable/limited direct absorption	Microbial biotransformation	↑ bioactive metabolite production	Interindividual variability, dysbiosis

FODMAPs, fermentable oligosaccharides, disaccharides, monosaccharides, and polyols; IBS, irritable bowel syndrome. Arrows indicate an increase (↑) or decrease (↓) relative to habitual or baseline conditions.

## Data Availability

Data sharing does not apply to this article as no datasets were generated or analyzed during the current study.
